# The effects of intergroup competition on prosocial behaviors in young children: a comparison of 2.5–3.5 year-olds with 5.5–6.5 year-olds

**DOI:** 10.3389/fnbeh.2015.00016

**Published:** 2015-02-12

**Authors:** Yi Zhu, Xian Guan, Yansong Li

**Affiliations:** ^1^School of International Business, Southwestern University of Finance and EconomicsChengdu, China; ^2^Social Work Center, Southwestern University of Finance and EconomicsChengdu, China; ^3^Reward and Decision-making Team, Cognitive Neuroscience Center, CNRS UMR 5229Bron, France

**Keywords:** prosocial behaviors, egalitarianism, parochial altruism, intergroup competition, cooperation, economic games, young children

## Abstract

Group-based competition is considered to be a ubiquitous social context in human society. However, little is known about its potential effects on children’s prosocial behaviors. To this end, we designed an experiment in which two age groups (2.5–3.5 years of age and 5.5–6.5 years of age) engaged in an intergroup competition task where they did a so-called “game” where each child transferred table tennis balls with a spoon from one container to the other. The non-intergroup competition condition was identical to the intergroup competition condition with one exception—no intergroup competition manipulation was involved. Then, they were required to perform two economic games used to measure their prosocial behaviors. We found that under the non-intergroup competition condition, as children aged, their behaviors tended to be more fairness-oriented (such as an increase in egalitarian behaviors). However, under the intergroup competition condition, children at 2.5–3.5 years of age tended to behave prosocially towards their ingroup members compared with those who are at 5.5–6.5 years of age. The behavioral pattern under the intergroup competition condition reflects strengthening prosocial tendencies driven by the intergroup competition in younger children and simultaneously weakening intergroup competition-driven prosocial tendencies possibly due to the development of fairness-oriented behaviors in older children. Taken together, these results point to the importance of considering the effects of competitive contexts on children’s social behaviors and may have important implications for further research on the role of competitive contexts in the development of human prosocial behaviors.

## Introduction

Prosocial behaviors refer to acts that are intended to help or benefit another individual or group of individuals (Eisenberg and Fabes, 1998). In our societies, these behaviors are highly valued and are one of the most important markers of competency in children. As a result, understanding the development of such behaviors has been gaining increasing attention over the past decades (Eisenberg and Fabes, 1998; Hay and Cook, 2007). Accumulating evidence indicates that prosocial behaviors have deep roots in human lifespan development. It has been revealed that prosocial behaviors emerge by the end of infancy in human ontogeny (Zahn-Waxler et al., 1992; Eisenberg and Fabes, 1998; Warneken and Tomasello, 2006; Warneken et al., 2007; Schmidt and Sommerville, 2011; Sloane et al., 2012). For example, young children between 12 to 18 months of age have begun to display prosocial behaviors, such as giving their toys to their parents, even without being reinforced by external praise (Dunfield et al., 2011). Moreover, the development of such behaviors continues as children gradually mature (Zahn-Waxler et al., 1992; Fehr et al., 2008; Bauer et al., 2014a; Ongley et al., 2014).

More importantly, advances in understanding of developmental trajectories in human prosocial behaviors also help understand the roles of contextual factors in shaping children’s prosocial behaviors. For instance, children’s social companions (de Guzman et al., 2008), parental care (Stewart and McBride-Chang, 2000; Bauer et al., 2014b), and cultural diversity (Rogof, 2003; Bronfenbrenner and Morris, 2006; Eisenberg et al., 2006; Chen and Eisenberg, 2012; House et al., 2013) have been shown to influence children’s prosocial behaviors. Among those contextual factors, intergroup competition is considered to be a ubiquitous social context in human society, which has been shown to play an important role in individuals’ behaviors (Stein, 1976; Bornstein, 2003; Simmel, 2010). For instance, empirical studies in adults reveal that intergroup competition enhances ingroup cohesion, whereby individuals behave prosocially towards members of their own group relative to outgroup members (Bornstein et al., 2002; Gunnthorsdottir and Rapoport, 2006; Blattman, 2009; Puurtinen and Mappes, 2009; Benard, 2012; Hugh-Jones and Zultan, 2012; Bauer et al., 2014a). As a result, group-based competition has been recognized as one of the most important driving forces in the evolution of human prosocial behaviors (Bowles, 2006). However, little is still known about the potential effects of intergroup competition on prosocial behaviors in children.

The present study aimed at exploring the possible effects of intergroup competition on children’s prosocial behaviors. To achieve this goal, we designed an experiment in which different age groups (2.5–3.5 years of age and 5.5–6.5 years of age) engaged in an intergroup competition task, which was followed by two economic games (a sharing game and an envy game) used to measure their prosocial behaviors. Specifically, in the intergroup competition task, two equal-sized groups under intergroup competition and non-intergroup competition conditions engaged in a so-called “game” where each child transferred table tennis balls with a spoon from one container to the other, and the number of balls successfully transferred by all members of each group within 20 s was counted. The non-intergroup competition condition was identical to the intergroup competition condition with one exception—no intergroup competition manipulation was involved. In the present study, this non-intergroup competition condition served as a benchmark condition because of unequal sample size between the intergroup competition and the non-intergroup competition condition.

## Materials and methods

### Participants

Participants were 328 preschoolers from 5 private preschools (Mai Ke Rui kindergarten, Havard Star kindergarten, Hai Xia Xin Cheng kindergarten, Liu Cheng Mei Yu kindergarten, and Duolaimi kindergarten in Chengdu, China). Seven of them were excluded from the final data analysis due to either their inability to follow decision rules (*n* = 3) or a potential confounding effect caused by the presence of their teachers during the experiment (*n* = 4). The remaining 321 participants were divided into 2 groups: 157 young children (2.5–3.5 years of age) and 164 old children (5.5–6.5 years of age). Due to the fact that gender difference in distributive behaviors have been observed in young children (Stewart and McBride-Chang, 2000; Ongley et al., 2014), the present study included almost half males (*n* = 162) and half females (*n* = 159) (Table 1). This research was approved by ethics committees of these 5 preschools.

**Table 1:** **Structure of the intergroup competition task**.

Condition	Outcome	Age groups	A	B	C	D	E	Total
Intergroup competition	Win	2.5–3.5	15	15	12	9	11	62
		5.5–6.5	14	10	12	12	15	63
		Total	29	25	24	21	26	125
	Loss	2.5–3.5	15	15	12	9	9	60
		5.5–6.5	14	12	12	12	15	65
		Total	29	27	24	21	24	125
Non-intergroup competition	–	2.5–3.5			12	12	11	35
		5.5–6.5			12	12	12	36
		Total			24	24	23	71

*A, B, C, D, and E denote five preschools, and numbers in each of the five columns are the number of participants in each experimental condition and each age group. Participants under the non-intergroup competition condition were only recruited from C, D, and E preschools. Seven participants were excluded from the final data analysis: In A, two participants were excluded due to the presence of their teachers. In B, one participant did not follow rules during the task, and one participant was excluded because of their teacher’s presence. In E, two participants were excluded due to their inability to follow the instruction and the other one participant was excluded because of their teacher’s presence*.

### Experimental procedure

#### Intergroup competition manipulation

The intergroup competition task included two conditions: an intergroup competition condition and a non-intergroup competition condition. Under the intergroup competition condition, participants (*n* = 250) engaged in an intergroup competition task, which has been widely employed to investigate the effects of group-based competition on human behaviors (Bornstein et al., 2002; Gunnthorsdottir and Rapoport, 2006; Halevy et al., 2008; Benard, 2012; Böhm and Rockenbach, 2013). One important feature of such intergroup competition manipulation in the present study is that it could result in two consequences (winning and losing) based on outcomes of such between-group rivalry, whereby both winning groups (*n* = 125) and losing groups (*n* = 125) can be formed accordingly. This allows us to additionally study effects of group-based winning and losing experiences on children’s prosocial behaviors in the present study.

More specifically, under the intergroup competition condition, six children from the same classroom were led by their teachers to a quiet room. They were randomly divided into two equal-sized groups. Following previous studies in which children’s group affiliation was identified using different colors (Bigler et al., 1997; Patterson and Bigler, 2006; Buttelmann and Böhm, 2014), colored ribbons (in red or blue) on children’s arm were used to mark their group affiliation (a red group and a blue group) in the present study. Afterwards, a female research assistant explained the “game” procedures to them. The explanation was repeated when necessary to ensure that all children fully understood the rules. Then, each group engaged in their teamwork simultaneously—each child transferred table tennis balls with a spoon from one container to the other, and the number of balls successfully transferred by all members of each group within 20 s was counted. Building on the outcomes of the between-group rivalry, each of these two groups could be identified to be either a winning or a losing group. Each member of the winning group was rewarded a sticker in front of the losing group who received no reward. These two competing groups engaged in their teamwork in the same room. During the competition, two female research assistants counted and kept records of the performance of each group respectively.

In contrast, the treatment under the non-intergroup competition condition (*n* = 71) was identical to the intergroup competition condition described above with one exception—no intergroup competition manipulation was involved. Specifically, six children from the same classroom were randomly divided into two equal-sized groups. Colored ribbons (in red or blue) on children’s arm were used to identify their group affiliation. Then, each group engaged in their teamwork simultaneously—each child transferred table tennis balls with a spoon from one container to the other, and the number of balls successfully transferred by all members of each group within 20 s was counted. In order to control for the potential implicit competition arising from the observation on other group’s performance, both groups could not see each other’s performance. Furthermore, no explicit information about the outcomes of between-group rivalry was delivered to each group in this control condition. Note that the sample size under the non-intergroup competition condition (*n* = 71) were not equal to that under the intergroup competition condition (*n* = 250). The unequal sample size between these two conditions makes it less sensitive to exploring the possible interaction effects between intergroup competition and age. In spite of this, the non-intergroup competition condition can still serve as an important benchmark condition against which participants’ behaviors can be compared and therefore, help illustrate effects of intergroup competition on children’s prosocial behaviors.

#### Economic games measuring children’s prosocial behaviors

To examine the effects of the intergroup competition on children’s prosocial behaviors, the present study adopted a so-called “choice paradigm” to measure how children made resource allocations between self and in-group partners. We used a sharing game and an envy game to measure children’s prosocial behaviors, since these two economic games have been widely used in previous studies investigating the developmental changes in human prosocial behaviors (Fehr et al., 2008; Warneken et al., 2011; Bauer et al., 2014a; Sheskin et al., 2014a).

**Figure 1:**
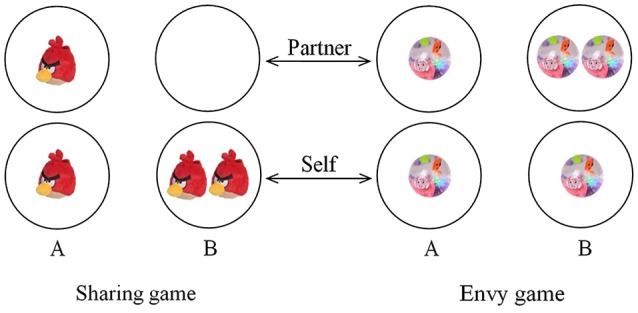
**Economic games employed in the present study**. Participants made choices in a sharing game and an envy game. Soft toys (Angry Bird) and flash water glitter balls (Happy Sheep) were used as distributional resources in these two games, respectively. Between-game differences in toys may help rule out satiation effects, and within-game homogeneity of toys may help control for individual differences in preferences for colors and styles which could potentially affect children’s decision-making behaviors.

Specifically, following the intergroup competition task, each participant was led by a research assistant to a different quiet room to do these two economic games. In the sharing game (Figure 1), participants chose between (1, 1)—one piece of toy for self and the other for an in-group partner—and (2, 0)—two pieces of toys for self and none for an in-group partner. Since the former personal payoffs was less than the latter one in the sharing game, choosing (1, 1) indicated that participants’ behaviors were mainly driven by their egalitarian motives, while choosing (2, 0) indicated that their behaviors were mainly driven by their proself motives. In the envy game (Figure 1), they could choose between (1, 1)—one piece of toy for self and the other for an in-group partner—and (1, 2)—one piece of toy for self and 2 for an in-group partner. In this game, the personal payoffs of the two choices were identical, but the sum of such payoffs in the latter choice (3 pieces) was larger than that in the former one (2 pieces). As a result, choosing (1, 1) in this game indicated that their behaviors were mainly driven by either their egalitarian motives or by their aversion to disadvantageous inequality, while choosing (1, 2) indicated that their behaviors were mainly driven by either their social welfare concerns or altruistic motives.

## Results

### Intergroup competition condition

#### The sharing game

**Figure 2:**
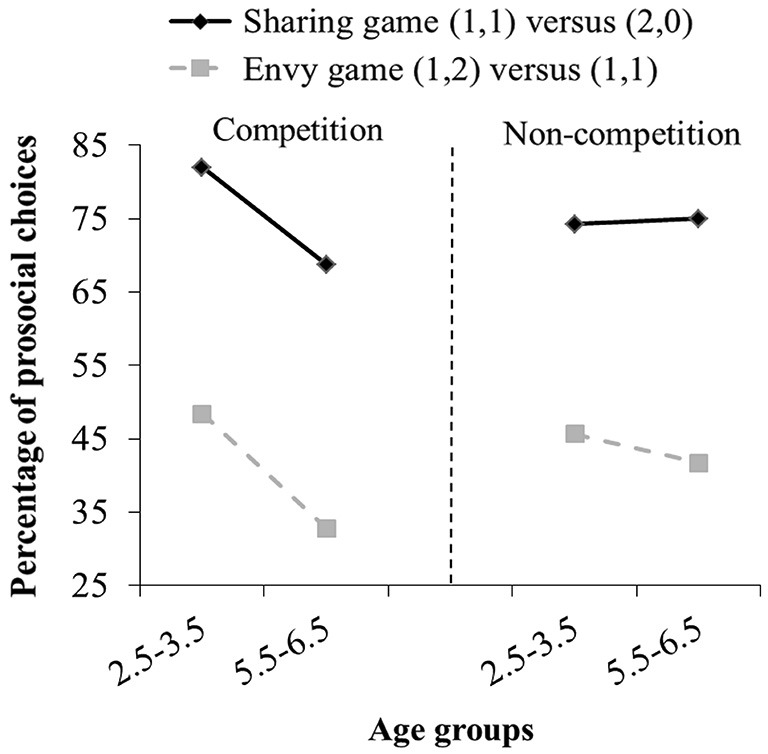
**The effects of intergroup competition on prosocial choices in two economic games**. The black solid lines denote the percentage of egalitarian choices (1,1) in the sharing game, while the gray dashed lines denote the percentage of altruistic choices (1,2) in the envy game. Under the intergroup competition condition and the non-intergroup competition condition, participants’ choices determine an ingroup member’s payoffs. Under the intergroup competition condition, the percentage of egalitarian choices (1,1) vs. selfish choices (2,0) in the sharing game decreases with age and the percentage of altruistic choices (1,2) vs. the egalitarian choices (1,1) also decreases with age in the envy game. Under the non-intergroup competition, the percentage of egalitarian choices increases with age, while the percentage of altruistic choices decreases with age, indicating that young children tend to be more egalitarian as their age increases.

Our data analysis on participants’ behaviors in the sharing game revealed that there were 75% of children who chose (1, 1), significantly above a chance level (50%) (Binomial test, *p* < 0.001, *n* = 250), suggesting that young children who experienced the intergroup competition behaved prosocially towards their in-group partners. This was further substantiated by our analyses on the egalitarian choices within each group (Binomial test: *p* < 0.001, *n* = 122, for 2.5–3.5 years of age; *p* < 0.001, *n* = 128, for 5.5–6.5 years of age). However, the effects of the winning and losing outcomes on egalitarian choices in this game were not statistically significant (Fisher’s exact test, *p* = 0.88, *n* = 250), 76% of children in the losing group and 74% of children in the winning group chose (1, 1). Neither did we observe a significant gender difference in egalitarian choices, because the frequencies of egalitarian choices by females (76%) and males (75%) were almost identical (Fisher’s exact test, *p* = 1.00, *n* = 250). More importantly, we observed a pronounced age effect: the frequency of egalitarian choices made by children at 2.5–3.5 years of age (82%) in this game was significantly higher than that made by their old counterparts (69%) (Figure 2) (Fisher’s exact test, *p* < 0.05, *n* = 250), indicating that children at 2.5–3.5 years of age tend to share their resources with their in-group partners after experiencing the intergroup competition. Finally, our statistical analysis did not reveal a significant interaction effect between competition outcomes (winning vs. losing) and age (Wald’s *χ*^2^ = 0.726, *p* > 0.05), indicating that the observed age effect on choice behaviors in the sharing game did not depend on the competition outcomes.

#### The envy game

In the envy game, 60% of our participants chose (1, 1), which was significantly different from the chance level of 50% (Binomial test, *p* < 0.005, *n* = 250), indicating that these children tried to avoid the disadvantageous inequality. Our subsequent analysis on each age group’s behaviors found that the frequency of egalitarian choices for children at 2.5–3.5 years of age was only 2% above the chance level (50%) (Binomial test, *p* = 0.79, *n* = 122). In contrast, the frequency of egalitarian choices for children at 5.5–6.5 years of age was 17% above the chance level (50%) (Binomial test, *p* < 0.001, *n* = 128). Because the option (1, 2) only increased their partners’ payoffs, these results suggested that children at 5.5–6.5 years of age could be more aversive to the disadvantageous inequality and thus tended to be more egalitarian than children at 2.5–3.5 years of age. This was further supported by the direct comparison of frequencies of the (1, 2) choice between these two age groups (48% for younger children, 33% for older children; Fisher’s exact test, *p* = 0.01, *n* = 250) (Figure 2). Moreover, the comparisons of the winning (42%) and losing (38%) outcomes on altruistic choices and such choices between females (40%) and males (41%) did not reveal significant effects (Fisher’s exact test; *p* = 0.61 for effects of the winning and losing outcomes, *p* = 1.00 for the gender differences; *n* = 250). Finally, our statistical analysis did not reveal a significant interaction effect between competition outcomes (winning vs. losing) and age (Wald’s *χ*^2^ = 0.469, *p* > 0.05), indicating that the observed age effect on choice behaviors in the envy game did not depend on the competition outcomes.

### Non-intergroup competition condition (the benchmark condition)

Given that the non-intergroup competition condition provides a benchmark against which the observations under the intergroup competition can be compared, we explored participants’ behaviors under this benchmark condition. Under the non-intergroup competition condition, the majority of participants were egalitarian in the sharing game. Specifically, 75% of the children chose (1, 1), which is significantly different from the chancel level (50%) (Binomial test, *p* < 0.001, *n* = 71). Moreover, our subsequent analysis on the age effect on egalitarian choices revealed that the frequency of egalitarian choices increased slightly with age (72% at 2.5–3.5 years of age and 76% at 5.5–6.5 years of age) (Figure 2), although the difference between these two age groups was not statistically significant. This observation is consistent with previous studies showing the frequency of egalitarian choices increases with age (Fehr et al., 2008). In the envy game, we found that the frequency (44%) of altruistic choices (1, 2) was below the chance level (50%), although it did not reach statistical significance (Binomial test, *p* = 0.342, *n* = 71). Moreover, our subsequent analysis on the age effect on the frequency of altruistic choices (1, 2) showed that the frequency of altruistic choices decreased with age (46% at 2.5–3.5 years of age and 42% at 5.5–6.5 years of age). This observation in the envy game is also consistent with previous studies showing that the frequency of altruistic choices decreases with age (Fehr et al., 2008), indicating that as children age, they tend to be more egalitarian during bargaining activities. Finally, gender differences in both the egalitarian choices (1, 1) in the sharing game (Fisher’s exact test, *p* = 1.00, *n* = 250) and altruistic choices (1, 2) in the envy game were not significant (Fisher’s exact test, *p* = 0.47, *n* = 250) (Figure 2).

### The comparison between the intergroup competition and non-intergroup competition condition (the benchmark condition)

In addition, we further performed two separate logistic regression analyses on prosocial behaviors in these two economic games. For the sharing game, the analysis revealed a significant main effect of age (Wald’s *χ*^2^ = 5.735, *p* < 0.05). However, a main effect of competition (Wald’s *χ*^2^ = 1.002, *p* > 0.05) and the interaction between competition and age (Wald’s *χ*^2^ = 1.496, *p* > 0.05) did not reach statistical significance. Our subsequent analysis revealed a tendency for 2.5–3.5 year-old children to behave more prosocially under the intergroup competition condition (82%) than under the non-intergroup competition condition (74%). In contrast, there was a tendency for 5.5–6.5 year-olds to behave less prosocially under the intergroup competition condition (69%) than under the non-intergroup competition condition (75%) (Figure 2). With regard to the envy game, the main effect of age was observed (Wald’s *χ*^2^ = 6.210, *p* < 0.05). However, the main effect of competition and interaction between age (Wald’s *χ*^2^ = 6.210, *p* > 0.05) and competition (Wald’s *χ*^2^ = 0.795, *p* > 0.05) did not reach any significance. Our subsequent analysis revealed a relatively weak tendency for 2.5–3.5 year-olds to behave more prosocially under the intergroup competition condition (49%) than under the non-intergroup competition condition (46%) and in contrast, a tendency for 5.5–6.5 year-old children to behave less prosocially under the intergroup competition (33%) than under the non-intergroup competition condition (42%) (Figure 2).

### Behavioral types

**Figure 3:**
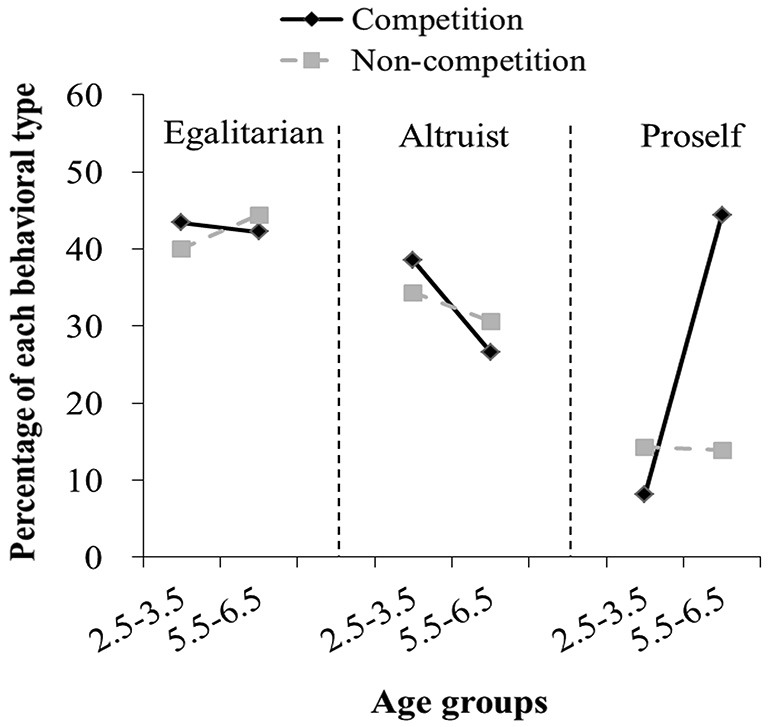
**The effects of intergroup competition on behavioral types**. The black solid lines denote the percentage of each of three behavioral types under the intergroup competition condition, while the gray dashed lines denote the percentage of each of these behavioral types under the non-intergroup competition condition. Under the intergroup competition condition, the percentage of egalitarians in children at 2.5–3.5 years of age is similar to that in children at 5.5–6.5 years of age, while under the non-intergroup competition condition, the percentage of egalitarians increases with age. It is obvious that the percentage of altruists is higher in younger children than in older children under the intergroup and non-intergroup competition conditions. The percentage of proselfs is significantly lower in younger children than in older children under the intergroup competition condition, but these behavioral type decreases with age under the non-intergroup competition condition.

If we took a between-game perspective, our participants’ behaviors could be classified into three different behavioral types (egalitarian, altruist, and proself) based on motives underlying their decisions in these two games, as described in previous studies (Fehr et al., 2008; Bauer et al., 2014a). Under the intergroup competition condition, the percentage of these three behavioral types across both groups was presented in Figure 3. Children who chose (1, 1) in both sharing and envy games were defined as egalitarians. Our analysis on this behavioral type showed that the percentage of egalitarians in children at 2.5–3.5 years of age (43%) was similar to that in children at 5.5–6.5 years of age (42%) (Fisher’s exact test, *p* = 0.90, *n* = 250). In contrast, with respect to altruists (those who chose (1, 1) in the sharing game and (1, 2) in the envy game), we found that there were more altruists in children at 2.5–3.5 years of age (39%) than in children at 5.5–6.5 years of age (27%) after experiencing intergroup competition (Fisher’s exact test, *p* < 0.05, *n* = 250). Finally, there were less proselfs (those who chose (2, 0) in the sharing game and (1, 1) in the envy game) in children at 2.5–3.5 years of age (8%) than in children at 5.5–6.5 years of age (25%) (Fisher’s exact test, *p* < 0.001, *n* = 250) (Figure 3). In addition, neither the winning and losing outcomes nor the gender had pronounced effects on these three behavioral types.

Under the non-intergroup competition condition, our analysis on the egalitarian behavioral type showed that the percentage of egalitarians increased with age (40% of children at 2.5–3.5 years of age and 44% of children at 5.5–6.5 years of age) (Figure 3), consistent with previous findings (Fehr et al., 2008; Bauer et al., 2014b). Similarly, with respect to altruists (those who chose (1, 1) in the sharing game and (1, 2) in the envy game), we found that there was a decreasing trend for children to be altruists as age increased (34% of children at 2.5–3.5 years of age and 31% of children at 5.5–6.5 years of age) (Figure 3). Finally, the percentages of proselfs (those who chose (2, 0) in the sharing game and (1, 1) in the envy game) in children at 2.5–3.5 years of age (14.3%) and in children at 5.5–6.5 years of age (13.9%) were almost identical (Figure 3).

## Discussion

Studying the development of human prosocial behaviors is critical to understanding large-scale cooperation in human society. Our analysis of children’s behaviors in the sharing game and envy game revealed that under the non-intergroup competition condition, children tended to be more egalitarian towards their ingroup members as their age increased, indicating the development of fairness-oriented behaviors with age. For one thing, this observation is consistent with a recent study showing that parochial altruism is already present in children of preschool age and increases with age (Fehr et al., 2008; Buttelmann and Böhm, 2014). For another, this finding is also consistent with previous findings showing that as children age, their behaviors supporting their interaction with others become more fair (Sheskin et al., 2014b). For instance, as children grow up, they tend to pay more attention to each member’s inputs and outputs of their groups and distribute resources based on group members’ efforts during the cooperative tasks (Leventhal et al., 1973; Lerner, 1974; Sigelman and Waitzman, 1991; McGillicuddy-De Lisi et al., 1994; Almås et al., 2010). The development of fairness-oriented behaviors has also been observed when children engage in bargaining activities. For instance, previous studies found that as children aged, they were more averse to disadvantageous inequality during bargaining activities (Murnighan and Saxon, 1998; Blake and McAuliffe, 2011; Sheskin et al., 2014a). This is corroborated by recent studies showing that as children aged, they tended to be more fair and egalitarian in economic games (e.g., Fehr et al., 2008) and to punish selfishness when they were involved in third party punishment (Jordan et al., 2014). Together, our observation under the non-intergroup competition adds to a growing literature on the development of strategic-oriented behaviors in children.

In contrast, under the intergroup competition condition, children at 2.5–3.5 years of age tended to behave prosocially towards members of their own group after experiencing the intergroup competition compared with children at 5.5–6.5 years of age. This seems to be at odds with the overall trend in the literature showing that as children age, they tend to be more egalitarian (Fehr et al., 2008). Why do young children behave differently between the intergroup competition and the non-intergroup competition? The preliminary observations below may provide some insights on the possible mechanisms underlying the role of competitive contexts in shaping children’s prosocial behaviors. For one thing, we found a tendency for 2.5–3.5 year-olds to behave more prosocially under the intergroup competition condition than under the non-intergroup competition condition, regardless of types of economic games (Figure 2). It has been argued that humans seems to be predisposed to be prosocial to other social members (Gintis et al., 2003; Bowles, 2006; Warneken and Tomasello, 2009) and therefore, this result seemed to reflect the enhanced prosocial instincts by the intergroup competition in younger children’s behaviors. For another, we also observed a tendency for 5.5–6.5 year-olds to behave less prosocially under the intergroup competition condition than under the non-intergroup competition condition, regardless of economic games (Figure 2). The weakening intergroup competition-driven prosocial tendencies may be due to the development of fairness-oriented behaviors in children. As a result, these results under the intergroup competition seemed to reflect the strengthening prosocial tendencies towards ingroup members driven by the intergroup competition in younger children and simultaneously the weakening intergroup competition-driven prosocial tendencies possibly due to the development of fairness-oriented behaviors in older children. Although our explanations about the possible mechanisms underlying the influence of competitive contexts in prosocial behaviors in young children need to be treated cautiously, the observations in the present study may still help stimulate further studies in this field.

Group-based competition is an ubiquitous nature of human groups (Stein, 1976; Simmel, 2010), and is viewed as one of the most important driving forces in the evolution of human prosociality (Bowles, 2006). Thus, studying effects of between-group rivalries (e.g., the relation between intergroup competition and cohesion) on children’s prosocial behaviors is of importance to understanding human social behaviors (Bornstein, 2003). The present study is among the few to study intergroup competition-cohesion relation from a developmental perspective. Although existing studies have documented how negative experiences (e.g., war, natural disaster) affected children’s prosocial behaviors (Wright, 1943; Li et al., 2013; Bauer et al., 2014a), evidence regarding effects of success or triumph on coping with adversity is rare. The unique advantage of the intergroup competition paradigm employed in the present study lies in that it not only facilitated our inquiry into the effects of strong group affiliation in preschoolers’ decision making, but also allowed us to study the full picture of behavioral consequences of both winning and losing outcomes arising from the intergroup competition.

Our observation that the frequency of prosocial behaviors in the winning groups was similar to that in the losing groups suggests that kindergartner’s prosocial behaviors were not sensitive to outcomes of the intergroup competition. This finding extends existing literature on the effects of intergroup competition or conflicts on the development of human prosocial behaviors. Collaboration among in-group members is one of prominent features of the intergroup competition paradigm adopted in the present study. Rewards were distributed evenly to group members who collaborated on winning the competition (i.e., each member was rewarded a sticker). Such setting is similar, if not identical, to the manipulation of collaborative outcomes observed in Warneken et al. (2011). Thus, the observation that young children in the winning groups behaved prosocially to in-group members is actually in line with findings from Warneken et al. (2011). In parallel, our observation that young children in the losing groups were also prosocial towards their in-group peers seemed to be congruent with observations in Wright (1943), indicating that frustration from experiences of loss in contests and situation where desirable toys were inaccessible may increase in-group cohesion among young children.

Culture could be another factor contributing to high frequency of egalitarian choices in the sharing game in our study. It has been shown that children growing up in collective cultures were more prosocial than those from individualistic cultures (Rao and Stewart, 1999; Rochat et al., 2009). Chinese children in the present study could be more inclined to viewing them embedded in close relations with peers, whereby they were more likely to consider in-group peers’ benefits.

Another factor that might also contribute to the observed age differences in prosocial behaviors is related to the One-Child Policy (OCP) introduced in 1979 to curb the population growth in China. OCP has been beneficial to the development of the country, but has also resulted in some issues (Hesketh and Zhu, 1997). One of them is concerned with the propensity of these children which is coined as “little emperor syndrome” (e.g., spoiled, egocentric). A recent experimental study indicated that the post-OCP children showed less interpersonal trust and risk-seeking than their pre-OCP counterparts (Cameron et al., 2013). Other studies also indicated that these only children were more self-centered than the sibling children in China (Jiao et al., 1986; Wang et al., 1998). Because the majority of participants in the present study involved such only children, one might speculate from socialization perspective that children grown up in this collectivistic culture could still consider less about others’ need in comparison with their younger counterparts. In line with this speculation, a recent study published in Chinese journal showed that the children’s offers in an ultimatum game decreased significantly with age (8, 11, 13, and 18 years of age) (Zhu et al., 2008). Whether such social policy as OCP caused age effects observed in our study is still an open question. As such, future efforts will be needed to figure out such age effects caused by the intergroup competition experiences so that we may have a clearer picture of the developmental patterns of prosocial behaviors in Chinese contexts.

The present study inevitably has some limitations: first, since this study was not conducted longitudinally, the observed differences may not reflect true developmental differences. Second, children’s prosocial behaviors are believed to be influenced by such factors as family incomes, parents’ education, and parenting styles, but we have not linked those variables to the performance observed in the present study due to privacy policy of these preschools. To better understand children’s prosocial behaviors, it will be worthwhile to devote resources and efforts to uncover how these factors influence the development of prosocial behaviors. Third, each child was led by a female research assistant to make decisions in economic games, though the third-party influence was held constant across individuals and experimental conditions, we still cannot rule out the possibility of third-party influence on children’s prosocial behaviors. Our next step would be to control for this factor by letting children to make decisions individually. Finally, only Chinese children were involved in the present study. It remains unclear whether our findings would generalize to other populations. This is an important question because cultural diversity (e.g., cultural individualism vs. cultural collectivism) has been shown to play an important role in prosocial behaviors in children (Rao and Stewart, 1999; Rochat et al., 2009). Thus, it would be interesting to take it into account in future research.

## Conclusion

In conclusion, we found that after experiencing the intergroup competition, children at 2.5–3.5 years of age tended to behave prosocially towards their ingroup members compared with those who are at 5.5–6.5 years of age. The behavioral pattern under the intergroup competition condition reflected the strengthening prosocial tendencies towards ingroup members driven by the intergroup competition in younger children and simultaneously the weakening intergroup competition-driven prosocial tendencies possibly due to the development of fairness-oriented behaviors in older children. Therefore, our results point to the importance of considering the effects of competitive contexts on the development of human prosocial behaviors. We think that the preliminary findings from our present study could shed light on the potential mechanisms underlying the role of competitive contexts in shaping children’s prosocial behaviors and thus may help stimulate further studies in this field.

## Conflict of interest statement

The authors declare that the research was conducted in the absence of any commercial or financial relationships that could be construed as a potential conflict of interest.
